# Genomic Sequences of Uropathogenic Escherichia coli Strains with Various Fluoroquinolone Resistance Profiles

**DOI:** 10.1128/MRA.00199-20

**Published:** 2020-09-17

**Authors:** Kayo Okumura, Masako Kaido, Eiki Yamasaki, Yasumasa Akai, Hisao Kurazono, Shingo Yamamoto

**Affiliations:** a Department of Veterinary Medicine, Obihiro University of Agriculture and Veterinary Medicine, Obihiro, Hokkaido, Japan; b Scientific Affairs, Sysmex Corp., Kobe, Hyogo, Japan; c Diagnostic Center for Animal Health and Food Safety, Obihiro University of Agriculture and Veterinary Medicine, Obihiro, Hokkaido, Japan; d Department of Urology, Hyogo College of Medicine, Nishinomiya, Hyogo, Japan; University of Maryland School of Medicine

## Abstract

The emergence of drug-resistant uropathogenic Escherichia coli (UPEC) has hampered antibiotic therapy for urinary tract infections. To elucidate the resistance mechanisms of UPEC, we performed whole-genome sequencing of eight UPEC strains with different fluoroquinolone resistance levels. Here, we report our sequencing data, providing a valuable resource for understanding such mechanisms.

## ANNOUNCEMENT

Escherichia coli is one of the most prevalent pathogens isolated from patients with urinary tract infections (UTIs) (1–4); in particular, uropathogenic E. coli (UPEC) causes over 85% of uncomplicated UTI cases (4, 5). Antibiotic therapy is the first-line strategy for patients with UTIs, and fluoroquinolones (FQs), such as levofloxacin (LVFX), are widely used for treating acute uncomplicated UTIs (6). However, due to increasing FQ usage, the prevalence of FQ-resistant E. coli isolates is increasing, complicating UTI antibiotic treatment. Many studies have attempted to detect mutations in quinolone resistance-determining regions (QRDRs) (7–10); the remaining genomic regions have not yet been thoroughly characterized. Therefore, to detect genome-wide mutations that confer resistance to FQs, we performed whole-genome sequencing of UPEC strains with various levels of resistance to LVFX.

The eight UPEC strains selected for whole-genome sequencing, with different levels of resistance to LVFX (Table 1), were developed in a previous study (11). Their LVFX-susceptible parental strains, GUC9 and GFCS1, were cultured on LB plates containing 0.5 to 64 μg/ml LVFX. After serial passage, we randomly selected colonies from the LVFX-containing plates and assessed their MICs against LVFX and their mutation profiles with respect to the QRDRs *gyrA*, *gyrB*, *parC*, and *parE* (11). Then, we selected strains with four different LVFX resistance levels (Table 1) from each parental strain. These strains (from frozen stocks) were streaked onto LB plates (Sigma-Aldrich, St. Louis, MO, USA) and grown overnight at 37°C. Next, a single colony was inoculated in brain heart infusion broth (Nissui, Tokyo, Japan) and incubated overnight at 37°C. Genomic DNA was extracted using extraction buffer (4 M guanidine thiocyanate, 200 mM NaCl, 100 mM Tris-HCl [pH 8.0], 25 mM EDTA, 1% 2-mercaptoethanol) and detergent buffer (10% [wt/vol] sodium *N*-lauroylsarcosine, 0.2% SDS, 200 mM NaCl) with 0.1-mm zirconia beads on a multibead shocker (Yasui Kikai, Osaka, Japan). After phenol-chloroform-isoamyl alcohol extraction, DNA was quantified using a Qubit 3.0 fluorometer and the Qubit double-stranded DNA (dsDNA) high-sensitivity (HS) buffer assay kit (Life Technologies, Burlington, Canada). DNA libraries were constructed using the Nextera XT DNA library preparation kit (Illumina, San Diego, CA, USA) according to the manufacturer’s instructions and sequenced using an Illumina MiSeq instrument for 600 cycles to produce 250-bp paired-end reads. Quality control checks on the sequence reads obtained were performed using FastQC v0.10.1 (https://www.bioinformatics.babraham.ac.uk/projects/fastqc) with default parameters. After sequence read verification using FastQC, trimming of poor-quality reads was deemed unnecessary. Illumina sequencing data were assembled with SPAdes v3.9.0 (Algorithmic Biology Lab, St. Petersburg Academic University of the Russian Academy of Sciences) using default parameters.

**TABLE 1:** General features of UPEC genomes determined by MiSeq sequencing in this study

Strain^*a*^	LVFX MIC (μg/ml)^*b*^	Mutation(s)	No. of reads	Coverage (×)	No. of contigs	Genome size (bp)	*N*_50_ (bp)	GC content (%)	BioSample accession no.	SRA accession no.
GUC9_S	0.5	*gyrA*, S83L	2,552,058	159	62	5,114,553	337,676	50.6	SAMD00203448	DRR207640
GUC9_L	2	*gyrA*, S83L	2,439,655	164	75	5,114,309	305,325	50.6	SAMD00203450	DRR207642
GUC9_I	8	*gyrA*, S83L	2,541,456	146	75	5,109,250	194,392	50.6	SAMD00203452	DRR207644
GUC9_H	128	*gyrA*, S83L; *gyrB*, E466D; *parE*: V466E	2,353,713	140	110	5,122,098	250,018	50.6	SAMD00203454	DRR207646
GFCS1_S	0.5	*gyrA*, S83L	2,315,729	136	75	5,110,960	184,144	50.6	SAMD00203449	DRR207641
GFCS1_L	2	*gyrA*, S83L	3,132,078	182	82	5,082,523	150,381	50.6	SAMD00203451	DRR207643
GFCS1_I	32	*gyrA*, S83L/D87G	2,139,959	121	110	5,086,017	114,120	50.6	SAMD00203453	DRR207645
GFCS1_H	128	*gyrA*, S83L/D87G; *parC*, S80I	3,630,647	193	78	5,078,104	150,811	50.6	SAMD00203455	DRR207647

a These strains were derived from two parental strains (GUC9 and GFCS1) with different levels of resistance to LVFX. _S, susceptible (<1 μg/ml); _L, low level of resistance (>2 μg/ml); _I, intermediate level of resistance (>4 to 32 μg/ml); _H, high level of resistance (>128 μg/ml).

b MICs for LVFX were determined in a previous study (11).

All mutations (nucleotide substitutions) in the QRDRs, i.e., *gyrA*, *gyrB*, *parC*, and *parE*, that were observed in a previous study (11) were found to be exactly the same in the sequenced LVFX-resistant strains (Table 1). This precise matching suggests that nucleotide variations in regions other than QRDRs confer drug resistance to FQs.

### Data availability.

This genome project was deposited in DDBJ/ENA/GenBank under the accession no. PRJDB9275. The sequencing data were deposited in the DDBJ Sequence Read Archive (DRA) under the accession no. DRA009585.
